# An examination of skeletal muscle and hepatic tissue transcriptomes from beef cattle divergent for residual feed intake

**DOI:** 10.1038/s41598-021-87842-3

**Published:** 2021-04-26

**Authors:** Clare McKenna, Kate Keogh, Richard K. Porter, Sinead M. Waters, Paul Cormican, David A. Kenny

**Affiliations:** 1 Animal and Bioscience Research Department, Teagasc Grange, Dunsany, C15 PW93 Co. Meath Ireland; 2 School of Biochemistry & Immunology, Trinity College Dublin, Dublin 2, D02 R590 Ireland

**Keywords:** Transcription, Transcriptomics

## Abstract

The selection of cattle with enhanced feed efficiency is of importance with regard to reducing feed costs in the beef industry. Global transcriptome profiling was undertaken on liver and skeletal muscle biopsies from Simmental heifers and bulls divergent for residual feed intake (RFI), a widely acknowledged feed efficiency phenotype, in order to identify genes that may be associated with this trait. We identified 5 genes (adj. p < 0.1) to be differentially expressed in skeletal muscle between high and low RFI heifers with all transcripts involved in oxidative phosphorylation and mitochondrial homeostasis. A total of 11 genes (adj. p < 0. 1) were differentially expressed in liver tissue between high and low RFI bulls with differentially expressed genes related to amino and nucleotide metabolism as well as endoplasmic reticulum protein processing. No genes were identified as differentially expressed in either heifer liver or bull muscle analyses. Results from this study show that the molecular control of RFI in young cattle is modified according to gender, which may be attributable to differences in physiological maturity between heifers and bulls of the same age. Despite this we have highlighted a number of genes that may hold potential as molecular biomarkers for RFI cattle.

## Introduction

Global agriculture is currently faced with the ambitious challenge of feeding a rapidly increasing global population, expected to peak at 9.2 billion by 2050^1^. This necessary increase in agricultural outputs must also be achieved within the current confines of arable land availability, thus it is essential that animal production systems become more efficient for the continued sustainability of the beef production sector. Animal feed can account for up to 75% of the variable costs in beef production systems; hence reduction of these costs is of importance^2^. Feed efficiency (dietary nutrient utilisation) in beef cattle is a trait of major economic importance^3^. Indeed, there is significant phenotypic and genetic variation among beef animals in their ability to convert dietary derived nutrients into saleable product^4–6^. Thus, by improving feed efficiency it is possible to reduce feed intake in cattle while still maintaining growth and skeletal muscle gain and ultimately contribute to beef production profitability and sustainability.

Residual feed intake (RFI), first described by Koch et al.^7^ is one such measure of feed efficiency, which can be defined as the difference between an animal’s actual versus its predicted feed intake based on average daily gain (ADG) and metabolic weight^8^. Through measuring an animal’s inherent RFI, feed-efficient animals which have a low-RFI value and consume less than expected as well as feed inefficient animals which have a high-RFI and consume more than expected value may be identified^3,9–15^. Indeed, it has been shown that growing beef cattle divergent for RFI can consume up to 20% less feed than their counterparts for the same level of performance^3,10,11,13–19^. This coupled with a moderate heritability estimate for RFI^4^ provides a feasible method for effective utilisation of this trait in production systems through genomic selection processes. However, although moderately heritable, a challenge remains to reliably and cost-effectively identify feed-efficient, low-RFI animals and to proliferate their genetics through animal breeding programmes. For example, the primary impediment to genetic progress and adoption of selection strategies based on RFI is both the large-scale logistics and expense of measuring individual animal feed intake and body weight.

In order to overcome the aforementioned limitations, studies have sought to uncover the underlying biology governing the trait, with the goal for the identification of molecular biomarkers^20^. This not only provides an attractive alternative to direct measurement of dietary intake on large numbers of animals^6^, but it also allows for a better understanding of the biological mechanisms underlying RFI which is important for progressing genomic selection. However although results in the literature have reported roles for biological processes including lipogenesis and the immune system toward variation in RFI primarily through transcriptional profiling, there is a distinct lack of commonality of key genes contributing to RFI across these studies. This is undoubtedly due to the multi-faceted nature of RFI as well as the contribution of factors including breed, gender, stage of development as well as production management and dietary intake test period length to the trait^20^. For example, a recent study that evaluated the molecular control of cattle divergent in RFI across three contrasting breed types, reported only 5 genes as commonly differentially expressed between high and low-RFI groups^21^. Furthermore, of these 5 genes only one, *SCD*, was differentially expressed in only one other molecular based RFI evaluation^22^. This lack of commonality, although due to confounding experimental designs represents a major short-fall toward the progress of genomics selection. Equally there is a dearth of information in relation to the potential effect of gender on subsequent variation in RFI, particularly under similar rearing conditions. Currently it is not known whether the same molecular mechanisms and genes are contributing to variation in RFI in heifers and bulls destined for beef production. Thus, the objective of this study was to evaluate the underlying biology regulating RFI through transcriptome sequencing in Simmental heifers and bulls reared under the same conditions from birth. It is estimated that two thirds of the variation in RFI is due to variation in resting energy expenditure^5^, with both muscle and liver representing important metabolic tissues with skeletal muscle accounting for over 50% body weight^12^ and liver accounting for 18–25% of total oxygen body consumption^23^. Thus, our efforts were focused towards examining the transcriptional alterations of these tissues between beef heifers and bulls divergent for high and low-RFI.

## Results

### Animal performance

Details of live weight gain, feed intake, and animal performance are presented in Table 1 for both bulls and heifers. Briefly, bulls and heifers had a mean ADG of 1.8 kg and 1.3 kg and dry matter intake (DMI) of 9.3 kg and 9.2 kg, respectively, during the RFI measurement period. Residual feed intake (kg DM/d) was 0.6 and − 0.7 for high RFI and low RFI bulls respectively, and 0.4 and − 0.3 for high RFI and low RFI heifers, respectively. Bulls and heifers ranked as high RFI consumed 10 and 15% more than their low RFI counterparts (p < 0.05), respectively. RFI ranking (high vs. low) did not affect (p > 0.05) initial bodyweight (BW), final BW or ADG, and this was consistent across gender.

**Table 1:** Summary of phenotypic data of bulls at end of feed intake trial.

Trait	High^1^(SD)	Low^1^(SD)	*P* value
**Bulls**			
No. of animals	5	5	˗
DMI, kg DM/d	10.1 (0.52)	8.4 (0.47)	0.02
RFI, kg DM/d	0.6 (0.05)	− 0.7 (0.04)	0.009
Metabolic bodyweight^0.75^, kg^2^	96.3 (5.19)	94.5 (7.82)	0.74
Initial bodyweight, kg	375.1 (32.35)	376.9 (34.44)	0.9
Final bodyweight, kg	511.8 (27.65)	496.6 (24.31)	0.62
ADG, kg/d	1.9 (0.33)	1.7 (0.29)	0.46
Backfatchange^3^, mm	1.5 (0.28)	1.5 (0.36)	0.63
**Heifers**			
No. of animals	5	5	˗
DMI, kg DM/d	9.8 (0.63)	8.6 (0.46)	0.03
RFI, kg DM/d	0.4 (0.05)	− 0.3 (0.02)	< 0.0001
Metabolic bodyweight^0.75^, kg^2^	91 (4.74)	92 (5.52)	0.89
Initial bodyweight, kg	368 (17.90)	361 (26.57)	0.73
Final bodyweight, kg	466 (18.58)	449 (27.28)	0.93
ADG, kg/d	1.3 (0.41)	1.4 (0.24)	0.59
Back fat change^3^, mm	1.3 (1.32)	1.2 (0.51)	0.16

^1^High RFI is inefficient and low RFI is efficient.

^2^Metabolic BW^0.75^, kg is determined as BW^0.75^ in the middle of the RFI measurement period which was estimated from the intercept and slope of the regression line after fitting a linear regression line through all metabolic BW (BW^0.75^) observations.

^3^Back fat change is mean of the fat depth at end of intake trial—mean of fat depth at start of intake trial.

### RNAseq read alignment and differential gene expression

**Figure 1:**
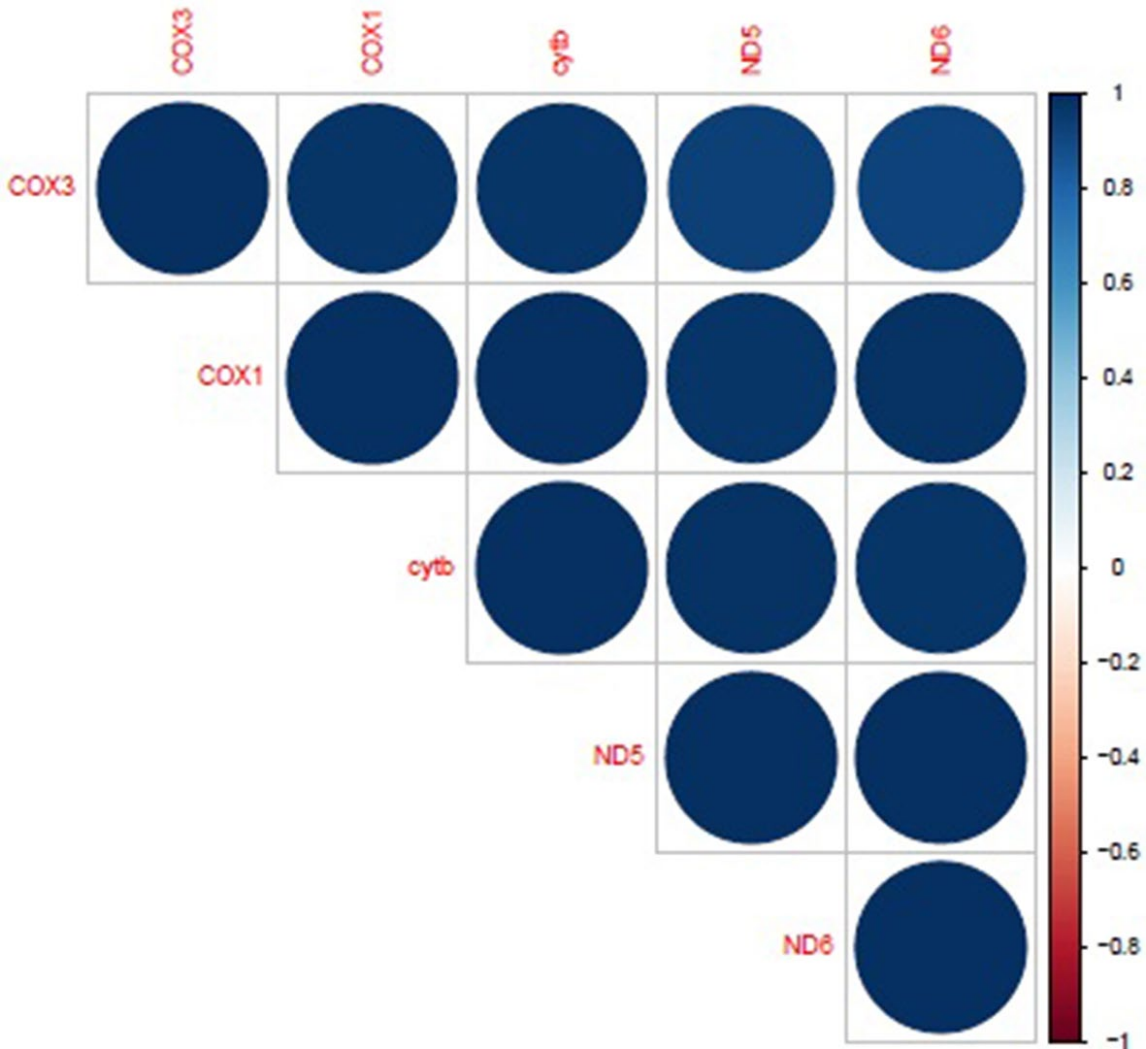
Correlogram highlighting the correlation between DEGs in skeletal muscle tissue from high and low RFI heifers. Visual representation of the correlation matrix highlighting the most correlated DEGs in skeletal muscle tissue from high and low RFI heifers. Blue circles (correlation value 1) indicate a high correlation between DEGs. Non-significant correlations are denoted as X. This image was generated through the use of the R (v3.4) Corrplot package^65^.

**Figure 2:**
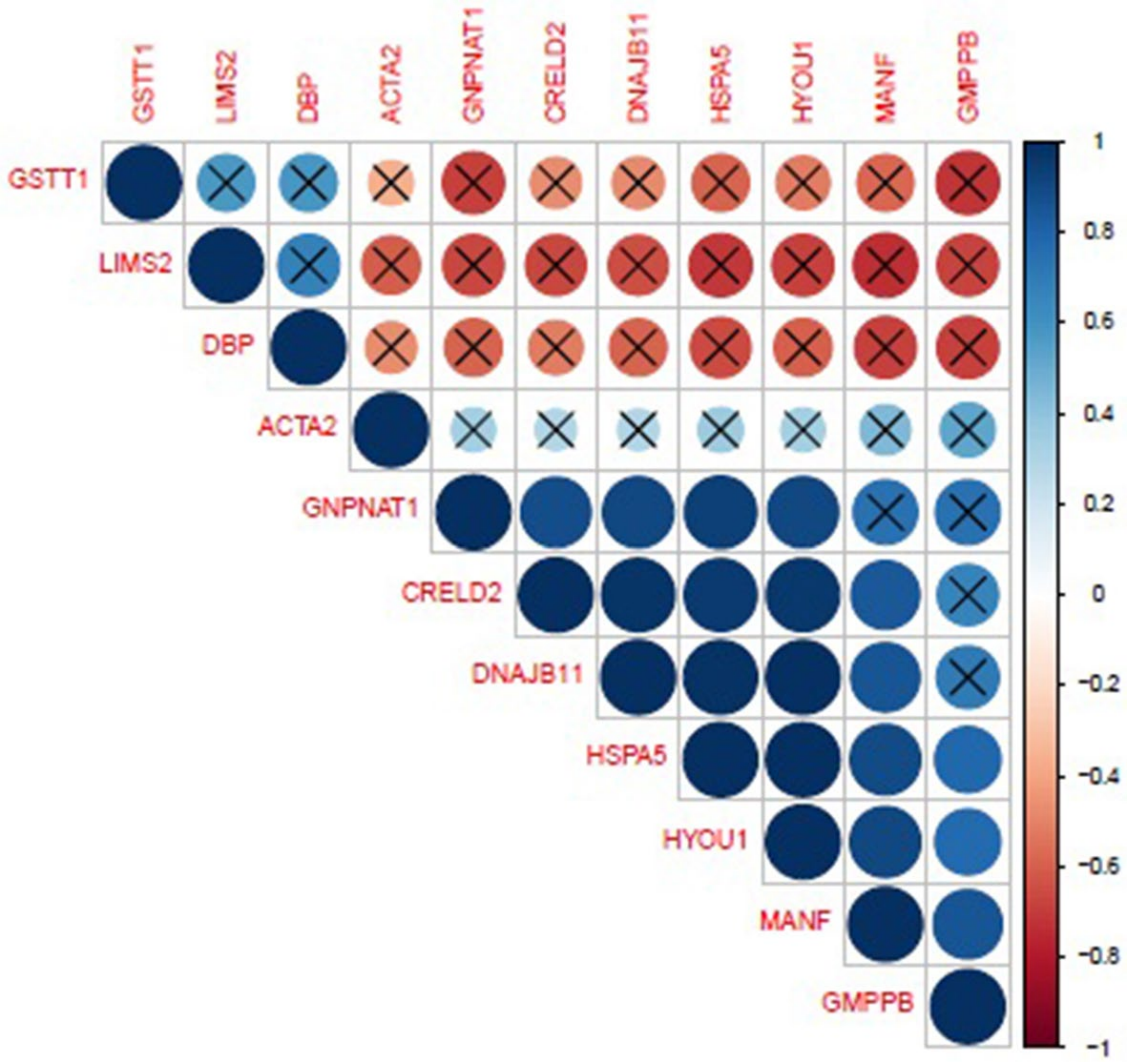
Correlogram highlighting the correlation between DEGs in skeletal muscle tissue from high and low RFI bulls. Visual representation of the correlation matrix highlighting the most correlated DEGs in hepatic tissue from high and low RFI bulls. Blue circles (correlation value 1) indicate a high correlation between DEGs while red circles (correlation value − 1) indicate no correlation between DEGs. Non-significant correlations are denoted as X. This image was generated through the use of the R (v3.4) Corrplot package^65^.

RNA sequencing data was successfully generated for all samples with approximately 22.4 million sequences per sample available across both tissues. On average approximately 85% of reads aligned to the bovine reference genome (UMD3.1) across both tissues analysed. The total number of genes expressed in each comparison was as follows: bull liver: 12,203 genes; bull muscle: 11,469 genes; heifer muscle: 10,766 genes and; heifer liver: 12,107 genes. A total of 5 genes (adj. p < 0.1) were identified as differentially expressed in skeletal muscle tissue between high and low RFI heifers with all 5 transcripts being up-regulated in the low RFI phenotype (Table 2). Figure 1 provides a visual representation of the correlation matrix pertaining to the differentially expressed genes (DEGs) from heifer skeletal muscle tissue, indicating all DEGs were highly correlated with each other. A total of 11 genes (adj. p < 0.1) were identified as differentially expressed in hepatic tissue between high and low RFI bulls (Table 3) with 8 transcripts being up-regulated and 3 being down-regulated in the low RFI phenotype. Figure 2 provides a visual representation of the correlation matrix pertaining to the DEGs from bull hepatic tissue, highlighting the most correlated genes. Correlation analyses between DEGs and RFI value for each heifer and bull group, identified mostly significant associations. For example, DEGs from the heifer skeletal muscle analysis were all significantly negatively associated (p < 0.05) with RFI, with the exception of *cytb*, which tended towards significance (p = 0.0622). Similarly within the liver analysis of bulls divergent for RFI, 7 of the 11 DEGs were significantly associated with RFI (p < 0.05), with *DBP*, *MANF* and *GMPPB* tending towards significance (p < 0.1). *ACTA2* expression was not significantly associated with RFI in the liver tissue of bulls (p = 0.3078). Correlation results between DEGs and RFI value for each group are presented in Table 4. None of the genes detected in heifer hepatic tissue and skeletal muscle tissue from bulls were found to be differentially expressed.

**Table 2:** Differentially expressed genes in the skeletal muscle of heifers divergent for RFI.

Ensemble Gene ID	Symbol	Log^2^FC^1^	adj. *P*-value
ENSBTAG00000043561	*COX1*	1.531959715	0.04229038
ENSBTAG00000043563	*ND5*	2.05811224	0.065711452
ENSBTAG00000043546	*ND6*	1.893462257	0.071589115
ENSBTAG00000043550	*cytb*	1.621906928	0.08104623
ENSBTAG00000043560	*COX3*	1.333918891	0.08240735

^1^ Log^2^Fold change was calculated for each gene from the expression value of low-RFI compared to high-RFI.

**Table 3:** Differentially expressed genes in the hepatic tissue of bulls divergent for RFI.

Ensemble Gene ID	Symbol	Log^2^FC^1^	adj. *P*-value
ENSBTAG00000007662	*HSPA5*	1.366758112	0.002345638
ENSBTAG00000006262	*LIMS2*	− 0.816790737	0.007056497
ENSBTAG00000047801	*CRELD2*	1.0379502	0.007056497
ENSBTAG00000010322	*HYOU1*	1.22672561	0.00984059
ENSBTAG00000031797	*MANF*	1.097562958	0.021052189
ENSBTAG00000000170	*GSTT1*	− 0.844108433	0.025190925
ENSBTAG00000014614	*ACTA2*	1.074998203	0.026112848
ENSBTAG00000003151	*DNAJB11*	0.910521574	0.026285489
ENSBTAG00000006754	*DBP*	− 1.022594741	0.044902486
ENSBTAG00000032026	*GMPPB*	0.632308333	0.054715662
ENSBTAG00000005344	*GNPNAT1*	0.619902591	0.07989897

^1^ Log^2^Fold change was calculated for each gene from the expression value of low-RFI compared to high-RFI.

**Table 4:** Correlation between DEGs and RFI value in both heifers and bulls.

Gene	RFI (*r)*	*P*-value
**Heifer (skeletal muscle)**		
*COX1*	− 0.73599	0.0238
*ND5*	− 0.73766	0.0233
*ND6*	− 0.70876	0.0326
*cytb*	− 0.64221	0.0622
*COX3*	− 0.7102	0.032
**Bull (liver)**		
*GSTT1*	0.7391	0.0146
*DNAJB11*	− 0.8317	0.0028
*GNPNAT1*	− 0.95164	< 0.001
*LIMS2*	0.68633	0.0284
*DBP*	0.6169	0.0768
*HSPA5*	− 0.85863	0.0015
*HYOU1*	− 0.80702	0.0048
*ACTA2*	− 0.35935	0.3078
*MANF*	− 0.62913	0.0513
*GMPPB*	− 0.62534	0.0532
*CRELD2*	− 0.81414	0.0041

### Differentially expressed genes and pathway analysis

Functional analysis of DEGs in hepatic tissue of bulls revealed amino sugar and nucleotide sugar metabolism as well as processes associated with the endoplasmic reticulum to be affected by divergence in RFI (Table 5). Functional analysis of heifer skeletal muscle DEGs indicated a role for oxidative phosphorylation and mitochondrial function towards divergence in RFI (Table 6). All DEG were successfully mapped to a molecular or biological pathway and/or category in the Ingenuity Pathway analysis (IPA) database. DEGs were analysed and separated according to their biological function within IPA. The top canonical pathways affected by RFI in heifer skeletal muscle tissue and hepatic tissue of bulls are presented in Table 7. These included enriched pathways related to mitochondrial function and oxidative phosphorylation in heifers and Aldosterone Signalling, GDP-mannose Biosynthesis, Nuclear factor erythroid 2-related factor 2 (NRF2) mediated oxidative stress response, and Eukaryotic Initiation Factor 2 (EIF2) signalling in bulls.

**Table 5:** Enriched GO functions in hepatic tissue from bulls divergent for RFI.

GO term	Adj. *P *value
Regulation of response to endoplasmic reticulum stress	0.00926
Endoplasmic reticulum chaperone complex	0.0006678
Endoplasmic reticulum lumen	0.0008020
Endoplasmic reticulum	0.001742
Protein processing in endoplasmic reticulum	0.0143
Amino sugar and nucleotide sugar metabolism	0.03819

**Table 6:** Enriched GO functions in skeletal muscle from heifers divergent for RFI.

Term	Adj. *P*-value
Oxidoreductase activity	4.571E−05
Cellular respiration	1.736E−05
Electron transport chain	2.094E−05
Oxidative Phosphorylation	0.002167
Mitochondrial membrane	9.812E−06
Mitochondrion	0.0006311
Mitochondrial protein complex	0.008394
NADH dehydrogenase activity	0.01009
Membrane protein complex	0.008394
Inner mitochondrial protein complex	0.001441
ATP metabolic process	0.01944

**Table 7:** Canonical pathways derived from IPA analysis associated with divergence for RFI in bull hepatic tissue and heifer skeletal muscle tissue.

Canonical Pathway	*P* value
*Bull (liver)*	
Aldosterone signalling in epithelial cells	2.11E−03
GDP-mannose biosynthesis	2.54E−03
NRF2-mediated oxidative stress	2.83E−03
ILK signalling	2.92E−03
EIF2 signalling	3.70E−03
*Heifer (skeletal muscle)*	
Mitochondrial function	3.18E−11
Oxidative Phosphorylation	3.26E−09

## Discussion

Residual feed intake is a prime feed-efficiency trait to target within beef production systems; this is not only due to the moderate heritability of the trait but also due to its independence from production traits used to calculate it^20^. Furthermore, data from our own group provides evidence that RFI is a repeatable trait in beef cattle^11^. However, although well suited toward genetic propagation through genomic selection, insight into the underlying genes governing the trait are conflicting across studies^20,24^. This is undoubtedly due to the multifaceted nature of the trait as well as the influence of both animal and management factors toward the RFI phenotype. Additional individual experimental parameters may also be contributing to the lack of consistency across results, including for example, the measurement of the RFI phenotype and the length of the dietary intake test period^25^. Furthermore, RFI calculated within a specific population, as is the case in RFI transcriptional profiling studies, only reflects natural variation within that specific population, thus the level of divergence may be quite variable across different studies. In addition to these limitations, genomic selection models do not differentiate on the basis of gender, thus if molecular biomarkers are to be successfully employed for a trait it is essential to determine whether biological processes and specific key genes are regulating economically important traits such as RFI across differing gender types. Thus again the objective of this study was to evaluate any potential effects of differing gender to the underlying biological mechanisms regulating variation in RFI in both Simmental heifers and bulls. The animals used in the current study were from a purebred, well-characterised herd, reared as a contemporary group from birth with similar genetics, thus permitting a more equitable comparison of the effect of phenotypic RFI ranking. Our evaluations were focused towards both the liver and skeletal muscle tissue, the metabolic activities of which are both essential for overall body homeostasis and efficiency of an animal, with both organs being highly abundant in mitochondria^26^. Muscle accounts for approximately 50% of body mass and 25% of basal metabolic activity of an animal and plays an important role in resting energy expenditure^27^. Additionally, the liver is a highly oxidative organ accounting for 18–25% of total oxygen body consumption that is responsible for metabolising lipids, proteins and carbohydrates into biologically useful molecules^23^. We hypothesised that due to the metabolic importance of these organs, that variation in feed efficiency and energy expenditure (measured here using RFI) is likely to be reflected in the transcriptome of tissue from these organs. Understanding the essential biological processes contributing to variation in RFI is critical to elucidating the genetic basis, for this trait.

Although the RFI values pertaining to the animals used in this study showed clear significant divergence between high and low-RFI groups in both heifers and bulls, we failed to identify any DEGs within the liver tissue of the heifers and the skeletal muscle tissue of the bulls. Similarly, other studies evaluating the molecular control of RFI divergence in crossbred steers reported no DEGs following correction for multiple testing^28–30^. Therefore, the main observation of the current study is the inconsistent effect of RFI across (1) gender and (2) tissue for DEG profiles despite a 10% and 15% difference in DMI between high and low RFI heifers and bulls, respectively, with no difference in ADG in the current study. While both genders were of similar age and were reared under the same conditions, it is apparent that there was some divergence in the actual physiological stage of development, as might be expected, between bulls and heifers at the time of sample collection. Similarly, in a targeted gene expression study, complementary to this current study, we observed a significant effect of gender on the expression of lipogenesis genes within the subcutaneous adipose tissue, this is despite there being no significant difference in subcutaneous fat measurements^31^. Typically, heifers display earlier physiological maturity when compared to bulls of the same age, thus the identification of a gender effect in an energy storage accretion tissue such as adipose tissue was perhaps unsurprising, however the potential effect of physiological age and stage of maturity on metabolic tissues at the same age in the current study was potentially unexpected. Our results indicate that it will be unlikely that the key genes derived from the tissues examined in this study will be accurate predictors of genetic potential for RFI across gender. However, it is possible that greater differences may have been apparent at the molecular level, had sample-size and sequencing depth been greater. Moreover, the low number of genes identified in the current study does not mean that other tissues or organs within the body may provide more comparable results and thus reliable biomarkers, based on animal age and not physiological age or stage of development. Despite this, we determined possible molecular mechanisms and biological functions influencing RFI in beef cattle within tissue and gender subgroups. These data highlight a relationship between RFI and the transcriptomic networks involved in mitochondrial function in the skeletal muscle of heifers and evidence for a relationship between RFI and the aldosterone signalling pathway and the NRF2 mediated oxidative stress pathway in hepatic tissue of bulls.

We identified 5 genes that were statistically differentially expressed in skeletal muscle of high and low RFI heifers. Although we did not identify large numbers of DEGs, the level of transcriptional differences are consistent with the findings of other previously published RFI based studies^32–34^. Among the 5 DEGs, all 5, *COX1 (cytochrome c oxidase subunit 1), ND5 (NADH-dehydrogenase 5), ND6 (NADH-dehydrogenase 6), CYTB (cytochrome b),* and *COX3 (cytochrome c oxidase subunit 3),* were up-regulated in low RFI heifers compared to their high RFI counterparts. Additionally, all of these genes were significantly associated with RFI, with the exception of *cytb*, which only tended towards a significant association, further indicating a role for these genes to RFI variation in the skeletal muscle tissue of heifers. Interestingly all of these genes are components of the electron transport chain (ETC) in the mitochondrial inner membrane and are of major importance to overall energetic efficiency. *ND5* and *ND6* are subunits of the enzyme complex NADH dehydrogenase (ubiquinone) or complex I of the ETC. *CYTB* is the main subunit of coenzyme Q: cytochrome c–oxidoreductase or complex III of the ETC. *COX1* is the main subunit of cytochrome c oxidase or otherwise known as complex IV of the ETC and *COX3 is* a transmembrane subunit of this same complex. Previous transcriptomic experiments have reported a relationship between DEGs related to the complexes of ETC and RFI^12,35^. For example, increased levels of *COXII* (complex IV) and NADH dehydrogenase subunits have been shown to be associated with efficient animals at both the protein and transcriptome level^12,35–38^. However, although genes of the ETC have previously been reported in relation to variation in RFI, this is the first report of an up-regulation of *COX1, ND5, ND6, CYTB* and *COX3* in skeletal muscle tissue of cattle of low vs. high RFI. The lack of commonality of specific key genes underlying processes such as ETC to RFI phenotype may be due to the differences in breed types employed across studies, with Simmental cattle utilised in the current study. For example, in a study examining the effect of various breeds on the molecular control of RFI in liver tissue, Mukiibi et al.^21^ recorded only 5 genes as commonly differentially expressed across three differing breed types, however an evaluation of the biological processes showed a clear commonality for the underlying biological control of RFI irrespective of breed, but with different key genes dependent on the breed type^21^.

Using gene ontology (GO) enrichment analysis, we identified important processes underlying feed efficiency variation in skeletal muscle of heifers. These included functions related to mitochondrial metabolism, in particular oxidative phosphorylation. Similarly, the top canonical pathways identified by IPA were mitochondrial function and oxidative phosphorylation. Taken together, these analyses highlight a greater capacity for mitochondrial function in the low RFI animals. The relationship between mitochondria and RFI has been addressed previously and mitochondrial dysfunction and oxidative stress have been implicated as contributing to variation in feed efficiency across varying species^12,35,39–42^. Mitochondria are highly dynamic organelles that are responsible for 90% of the energy production in the body and are major reactive oxygen species (ROS) regulators^43^. It seems likely that variation in mitochondrial function could contribute to variation in energy utilisation. Furthermore, it has been demonstrated that feed efficient animals exhibit greater capacity to modulate conditions of oxidative stress^44^. Feed efficient animals have been shown to have a higher activity of all enzymes of the ETC across multiple species including broilers and lambs^39,40,42^ and the present study focused on cattle is in agreement with this. The DEG in the current study encode proteins involved in Complexes I, III and IV of the ETC indicating an impaired oxidative phosphorylation system in the skeletal muscle of the less efficient heifers. These results complement previous research suggesting an association between decreased respiration capacity and increased ROS production in less efficient animals^40,45^. Moreover, the results of the present study are reinforced by the observation of Kong et al.^38^ in which the mitoproteome was skewed towards high feed efficiency birds despite no difference in mitochondrial DNA between phenotypes, suggesting an increase in mitochondrial activity in the high feed efficiency phenotype^38^.

Within the hepatic tissue of bulls we identified 11 DEGs, 8 of which were up-regulated in the low RFI animals. These included; *HSPA5 (78 kDa glucose –regulated protein precursor)*, *CRELD2 (cysteine rich with EGF like domains 2)*, *HYOU1 (hypoxia up-regulated protein 1 precursor)*, *MANF (mesencephalic astrocyte derived neurotrophic factor)*, *ACTA2 (actin, alpha2, smooth skeletal muscle, aorta)*, *ENSBTAG00000003151 (DNAJ heatshock protein family (Hsp40) member B11, GMPPB (GDP-mannose pyrophosphorylase B)*, *GNPNAT1 (glucosamine-phosphate N-acetyltransferase 1)*. Three genes were down-regulated in the low RFI bulls. These included; *LIMS2 (LIM zinc finger domain containing 2)*, *GSTT1 (glutathione S-transferase theta 1)*, *DBP (D-box binding PAR bzip transcription factor)*. *HSPA5*, *CRELD2*, *HYOU1*, *ACTA2*, *DBP, MANF,* and *GSTT1* have previously been implicated in variation in feed efficiency^21–23,33,35,44,46–49^ and are potential candidate biomarkers for this complex trait. Furthermore, *HSPA5*, *CRELD2*, *HYOU1* and *GSTT1* were all significantly associated and *MANF* tended towards a significant association with RFI phenotype further implicating the importance of these key genes to RFI, not only to the bulls used in the current study but to other cohorts of cattle divergent for RFI status. Gene ontology analysis of hepatic tissue in bulls identified terms including those related to amino sugar and nucleotide sugar metabolism as well as others related to endoplasmic reticulum protein processing as significantly enriched. Additionally, Ingenuity pathway analysis identified Aldosterone Signalling in Epithelial cells, GDP-mannose Biosynthesis, NRF2 mediated Oxidative Stress Response and EIF2 Signalling as the top canonical pathways related to RFI in bull hepatic tissue. The results of the GO enrichment and IPA analysis indicate that oxidative response, protein processing and cell signalling in the liver are likely to be processes that are influencing variation in feed efficiency.

The aldosterone signalling pathway was identified by IPA as the top canonical pathway due to the up-regulation of the genes *HSPA5* and *DNAJB11* in the hepatic tissue of low RFI bulls. Aldosterone is secreted by the adrenal glands and has a major role in electrolyte and fluid homeostasis. The aldosterone signalling pathway has been implicated in feed efficiency previously in the spleen of inefficient animals^30^ and, interestingly, a GWAS analysis with cattle identified this pathway to be associated with variation in feed conversion ratio^50^. The protein encoded by *HSPA5* is a member of the *HSP70* family and as this protein interacts with many endoplasmic reticulum (ER) proteins it is likely to be important in monitoring protein transport through the cell^51^. *DNAJB11* as a member of the DNA-J family of proteins is involved in the correct folding of proteins^52^. Specifically DNAJB11 is involved in protein processing and metabolism of proteins and serves as a co-chaperone for *HSPA5* in the ER^49^. One of the many functions of this family of proteins is to stabilize new proteins by ensuring correct folding or by helping refold proteins that have already been damaged by cellular stress. Both *DNAJB11* and *HSPA5* have been implicated with feed efficiency previously^23,30,44,53^. In agreement with the current study, other work from our group^54^ observed that animals undergoing compensatory growth with concomitant improvements in feed efficiency have a higher hepatic transcript abundance of *DNAJB11* and *HSPA5*. Similarly, an up-regulation of *HSPA5* was observed in the hepatic tissue of low RFI animals by Paradis et al.^33^. Taken together these results are indicative of a greater capacity in controlling cellular function and organisation as well as protein metabolism in more feed efficient animals.

NRF2 mediated oxidative stress response was also observed to be an overrepresented pathway in the hepatic tissue of high RFI bulls in the current study due to the up-regulation of the genes *GSTT1* and *DBP*. NRF2 is a member of the cap ‘n’ collar basic region leucine zipper (cnc bZip) group of transcription factors^55^. This transcription factor is ubiquitously expressed in tissues but is only activated in response to a range of oxidative and electrophilic stimuli including ROS, antioxidants, glucose induced oxidative damage, heavy metals, and certain disease processes^55–57^. This canonical pathway has previously been associated with feed efficiency in a number of studies^28,48,49,53,58^. *GSTT1* is a member of the glutathione *S*-transferase family and is involved in metabolism of xenobiotics and in catalysing reactions between the antioxidant glutathione and a host of potentially toxic compounds, highlighting it as an important homeostatic molecule^59^. The glutathione S-transferase family has previously been implicated in feed efficiency in various species^28,48,49,53,58^. In agreement with the current study, Chen et al.^49^ and Lindholm-Perry et al.^28^ observed an up-regulation of these genes in feed inefficient cattle. *DBP* is a protein coding gene and amongst its cited functions is activation of circadian gene expression. Gene ontology annotations related to this gene include transcription factor activity, sequence-specific DNA binding and transcriptional activator activity and RNA polymerase II core promoter proximal region sequence-specific binding^60^. Additionally, *DBP* was also implicated as contributing to variation in RFI phenotype through the network analysis reported by Weber et al.^22^. Similarly, it has been shown that mice with increased FE have a lower expression of *DBP*^61^ which is in agreement with the present study. Taken together, our work and that of the aforementioned authors, suggest that less efficient animals are exhibiting an increased oxidative stress, reflected in their increased anti-oxidation activities.

The differential expression and significant association of *HSPA5, CRELD2, HYOU1, GSTT1* and *MANF* with RFI in the present study are also noteworthy as all six genes have been previously observed as differentially expressed in relation to feed efficiency in cattle^21,22,44,49,62^. While the biological significance of these genes in relation to feed efficiency remains unclear, due to their consistent presence in the literature, they should not be ruled out as potential biomarkers for this trait.

## Conclusion

The present study contributes to the published knowledge base regarding the transcriptomic regulation of variation in feed efficiency. Our work, in combination with that of others as previously mentioned, highlights common genes underpinning feed efficiency in cattle, as measured by RFI, regardless of breed or genetic background. RNA-seq analysis is an exploratory approach that provides new hypotheses to be further investigated by other complementary approaches including global proteomics and ultimately, potential variation in the genes identified in this study may provide a basis for the selection of candidate biomarkers for the RFI trait and, following appropriate validation, contribute to genomic selection breeding programmes to improve feed efficiency in beef cattle. However, the key message from this work highlights the inconsistency in gene expression profiles across genders of the same genetic merit. While this inconsistency may be explained by the differences in physiological maturity of the two genders, it indicates that extensive further investigation is required before biomarker selection for RFI can be adopted.

## Methods

All procedures involving animals in this study were conducted under an experimental licence from the Health Products Regulatory Authority in accordance with the cruelty to Animals Act 1876 and the European Communities (Amendment of Cruelty to Animals Act 1876) Regulation 2002 and 2005.

### Animal model

The animals used in this study were derived from a purebred herd of Simmental cattle originally established to examine various aspects of the biological control of the RFI trait and which has been well characterised to date in the published literature^13–19^. Briefly, purebred Simmental beef cattle derived from a herd phenotypically ranked on RFI were used for the current study^31^. Based on RFI phenotype the highest (n = 20) and lowest (n = 20) ranking cows and heifers were bred to pedigree Simmental sires with estimated breeding values for high and low RFI (see Crowley et al.^4^), respectively, through artificial insemination and multiple ovulation/embryo recovery technologies. There was no crossover of sires used across both cohorts of donor females. Resultant embryos were transferred to crossbred beef heifer recipients and the resulting calves (bulls, n = 16; heifers n = 18) were used for the current study. Pregnant heifers were managed under standard protocols and following calving were allowed to suckle their calves for a period of up to 7 days. In order to standardise rearing, calves were then abruptly weaned and were subsequently reared on an electronic calf feeder. Briefly calves were offered milk replacer (MR; Blossom Easymix; Volac, Co. Cavan, Ireland) and concentrate in pelleted form using an electronic feeding system (Vario; Foster-Tecknik, Engen, Germany), which recorded all feed-related events including intake of both MR and concentrate, drinking speed, as well as number of rewarded (when calves receive milk) and unrewarded (no milk dispensed) visits to the machine. Calves were subsequently weaned at 10 weeks of age and were offered concentrate and hay on a 50:50 dry matter basis until turnout to pasture at approximately six months of age. At approximately 15 months of age all cattle (n = 34) were housed within pens of between 5–7 animals/pen in a slatted floor shed. Cattle were fed once daily (0800 h) and were offered ad libitum concentrate (860 g/kg rolled barley, 60 g/kg soya bean meal, 60 g/kg molasses and 20 g/kg minerals/vitamins) and 3 kg grass silage to retain ruminal function. The animals had an acclimatisation period of 14 days to the ad libitum regime and test facilities before the experimental recoding period commenced. Feed intake was recorded daily and BW was recorded twice weekly. The recording period lasted 70 days. Concentrates and silage offered were sampled three times weekly and samples were stored at − 20 °C pending laboratory analysis. Samples of concentrates and silage were subsequently pooled on a weekly basis for DM determination. Concentrate samples were dried in an oven with forced-air circulation at 98 °C for 16 h for DM determination and forage samples dried at 40° for 48 h.

### Statistical analysis

Average daily live weight gain during the RFI measurement period for each animal was computed as the coefficient of the linear regression of BW (kg) on time (d) by using the GLM procedure of SAS 9.1 (SAS Inst. INC., Cary, NC). Mid-test metabolic BW (MBW) was represented as BW^0^^.75^ 35 d before the end of the test which was estimated from the intercept and slope of the regression line. Residual feed intake was calculated for each animal as the difference between actual DMI and expected DMI. Expected DMI was computed for each animal using a multiple regression model, regressing DMI on MBW, ADG and mean lumbar BF change. Animals were ranked within gender and following a power analysis utilising RFI phenotype data previously generated by our group^13–19^, a total of 5 animals per group was required for statistical analysis, thus the most extreme animals for high RFI (n = 5) and low RFI (n = 5) in each heifer and bull group were selected for further analysis.

### Biopsy sample collection

*M.longissimus thoracis et lumborum* (skeletal muscle) biopsies were harvested as described by Kelly et al.^12^ and hepatic tissue was collected by percutaneous punch biopsy as described by McCarthy et al.^63^ from animals deemed high and low RFI under local anaesthetic (5 mL Adrenacaine, Norbrook Laboratories (Ireland) Ltd.) at the end of the RFI measurement period. All surgical instruments used for tissue collection were sterilised and treated with 70% Ethanol and RNaseZap (Ambion, Applera Ireland, Dublin, Ireland). *M.longissimus thoracis et lumborum* biopsies were snap frozen in liquid nitrogen directly after collection and hepatic tissue biopsies were washed in sterile phosphate buffered saline and snap frozen in liquid nitrogen. All samples were subsequently stored at − 80 °C for long-term storage pending further processing.

### RNA isolation and purification

Total RNA was isolated from 50 mg of biopsy samples using QIAzol (Qiagen, UK). Tissue samples were homogenised in 1 mL of QIAzol reagent using a rotor-strator tissue lyser (Qiagen, UK) and chloroform (Sigma-Aldrich Ireland, Dublin, Ireland). RNA was subsequently precipitated and purified using the RNeasy plus Universal kit (Qiagen, UK) according to the manufacturer’s guidelines, which included a step to remove any contaminating genomic DNA. The quantity of the RNA isolated was determined by measuring the absorbance at 260 nm using a Nanordrop spectrophotometer ND-1000 (Nanodrop Technologies, Wilmington, DE, USA). RNA quality was assessed on the Agilent Bioanalyser 2100 using the RNA 6000 Nano Lab Chip kit (Agilent Technologies Ireland Ltd., Dublin, Ireland). RNA quality was also verified by ensuring all RNA samples had an absorbance (A260/280) of between 1.8 and 2.0 and RIN (RNA integrity number) values of between 8 and 10 were deemed high quality. Any samples that had an (A260/280) absorbance of less than 1.8 were cleaned using Zymo Research RNA clean & concentrator kit (Cambridge Biosciences, UK). High quality RNA samples were selected for cDNA synthesis.

### cDNA library preparation and sequencing

cDNA libraries were prepared from high quality RNA following the manufacturer’s instructions using the Illumina TruSeq mRNA sample prep kit (Illumina, San Diego, CA, USA). For each sample, 1 μg of RNA was used for cDNA library preparation. Resultant cDNA libraries were validated on the Agilent Bioanalyser 2100 using the DNA 1000 Nano Lab Chip kit. cDNA concentration was assessed using Nanordrop spectrophotometer ND-1000 (Nanodrop Technologies, Wilmington, DE, USA) and samples with > 25 ng/μl were deemed suitable for further analysis. Following quality control procedures, individual RNAseq libraries were pooled based on their respective sample-specific 6 bp adaptors and sequenced at 100 bp/sequence single-end read using and Illumina HiSeq 2500 sequencer. Approximately 22.4 million sequences per sample were generated.

### RNAseq data analysis

FASTQC software (v0.11.5) was used to check the quality of the raw sequencing reads. Input reads were then aligned to the bovine reference genome (UMD3.1) using STAR (v2.5.1). HTSeq (v0.6.1p2) software was used to calculate the number of sequenced fragments overlapping all protein-coding genes from the ENSEMBLv88 annotation of the bovine genome. The number of counts of reads mapping to each annotated gene from HTSeq was then collated into a single file and used for subsequent differential gene expression. The R (v3.4) Bioconductor package, EdgeR (v3.5)^64^, which uses a negative binomial distribution model to account for both biological and technical variability, was applied to identify statistically significant DEGs. The analysis was undertaken using moderated tagwise dispersion. An adjusted p value < 0.1 (Benjamini–Hochberg adjustment) was applied as a threshold to call genes with differential expression levels. The R (v3.4) Corrplot package^65^ was used to visualise the correlation matrices pertaining to the DEGs passing multiple correction for each comparison. Gene expression results for DEGs identified through transcriptional profiling were also correlated with RFI value, to determine potential associations between the DEGs and RFI. Correlations were undertaken using the CORR procedure in SAS 9.1 (SAS Inst. INC., Cary, NC).

### Gene ontology and pathway analyses

Biological processes, cellular components and molecular functions that were associated with the DEGs were identified using the g:Profiler GO enrichment tool^66^. The gene name and associated p-value were used as input for gene ontology and pathway analyses. Fisher’s exact test was used to determine the enrichment of the GO terms. *P-*values associated with each annotation term inside each cluster are Fisher Exact/EASE Scores. To examine the molecular functions and genetic networks; the RNAseq data was further interrogated using IPA (Ingenuity Systems, Redwood City, CA; http://www.ingenuity.com), a web based software application that enables identification of over-represented biological mechanisms, pathways and functions most relevant to experimental datasets or genes of interest^67^. Data were imported in a flexible format using the gene symbol as the identifier.

### Ethics declarations

All procedures involving animals in this study were conducted under an experimental licence from the Health Products Regulatory Authority in accordance with the cruelty to Animals Act 1876 and the European Communities (Amendment of Cruelty to Animals Act 1876) Regulation 2002 and 2005. All procedures described in this study were carried out in compliance with the ARRIVE guidelines.

## Data Availability

The datasets generated and analysed during the current study are available in the National Centre for Biotechnology Information (NCBI), Gene Expression Omnibus repository, and are accessible through GEO accession number GSE112793 [https://www.ncbi.nlm.nih.gov/geo/] under accession number.
